# Immunity to Soil-Transmitted Helminths: Evidence From the Field and Laboratory Models

**DOI:** 10.3389/fimmu.2020.01286

**Published:** 2020-06-23

**Authors:** Stefano A. P. Colombo, Richard K. Grencis

**Affiliations:** ^1^Department of Tropical Disease Biology, Liverpool School of Tropical Medicine, Liverpool, United Kingdom; ^2^Division of Infection, Immunity and Respiratory Medicine, Wellcome Trust Centre for Cell Matrix Research, Lydia Becker Institute for Immunology and Inflammation, The University of Manchester, Manchester, United Kingdom

**Keywords:** *trichuris muris*, trickle infection, Th2 immunity, *Heligmosomoides bakeri*, mucosal immunology, helminths, parasitism

## Abstract

Infection with soil-transmitted helminths (STH) remains a major burden on global health and agriculture. Our understanding of the immunological mechanisms that govern whether an individual is resistant or susceptible to infection is derived primarily from model infections in rodents. Typically, experimental infections employ an artificially high, single bolus of parasites that leads to rapid expulsion of the primary infection and robust immunity to subsequent challenges. However, immunity *in natura* is generated slowly, and is only partially effective, with individuals in endemic areas retaining low-level infections throughout their lives. Therefore, there is a gap between traditional model STH systems and observations in the field. Here, we review the immune response to traditional model STH infections in the laboratory. We compare these data to studies of natural infection in humans and rodents in endemic areas, highlighting crucial differences between experimental and natural infection. We then detail the literature to date on the use of “trickle” infections to experimentally model the kinetics of natural infection.

## Introduction

Soil-transmitted helminths (STH) are a highly diverse group of parasites present across the globe. Chronic life-long infection with at least one species of STH is common for most vertebrates (1). This includes humans and livestock in low to middle income countries (LMIC). The morbidity and reduced fitness associated with infection make STH helminthiases a major concern both for global health and for agriculture in endemic areas (2, 3). The infectious stages of these parasites are abundant in the environment and, due to their robustness against environmental insult, can persist there for long periods. The longevity of these parasites is compounded by their capacity to act as potent immunomodulators of their hosts (4).

A key determinant in the relationship between a host and STH parasite is the host's immune response. The host must balance an effective response to the parasite with limiting potentially detrimental immunopathology and exhausting vital resources (5). Similarly, the parasite must promote an immune response in the host that supports its own survival but that also protects the host from excessive pathology and infection by other potential pathogens. Given this, it is highly likely that anti-parasite immune responses have evolved to limit parasite burden and promote wound repair rather than to cause rapid and total parasite expulsion.

The majority of studies on immune responses to STHs are performed using rodent-specific STHs that have been adapted to the laboratory setting. These include the gastrointestinal (GI) nematodes, *Trichuris muris, Heligmosomoides sp*. (Formally *Nematospiroides dubius*. Herein we refer to the laboratory strain as *H. bakeri* and those identified in wild rodents as *H. polygyrus*. It should be noted, however, in the literature to date these names have been used interchangeably for experimental infections)*, Trichinella spiralis* and *Nippostrongylus brasiliensis*. Additionally, in some cases, human-specific species can be experimentally modeled in rodents, for example *Necator americanus* (6–9) and *Ancylostoma ceylanicum* (10, 11) albeit with limited success. Further, the larval migration that occurs during ascariasis, and hookworm infection, can be modeled in mice using the porcine STH, *Ascaris suum* (12).

Traditional experimental infections using well-established models typically rely on infecting mice with a single, artificially high dose of parasites. This is in contrast to the natural scenario in which frequent low-level exposures are likely to be more common. There is also a clear difference between the kinetics of traditional experimental infections and those seen in naturally infected populations. Thus, should we wish to fully understand the nuances of STH infection, there is a need to ensure that we are accurately modeling the natural situation.

## Infection in the Laboratory

Whilst there are species-specific responses based on the model STH used, many aspects of the immune response to experimental high-dose infections can be generalized. Upon invasion of the host, and often throughout infection, STHs cause considerable damage to tissue surrounding the site of infection. Migration of *N. brasiliensis* through the lung causes gross changes in tissue architecture and long term damage (13). Likewise, invasion of the gut epithelium and lamina propria, by *T. muris* and *H. bakeri*, respectively, causes considerable remodeling of the intestinal environment (14, 15).

Breaches by STHs at these barrier sites are associated with the release of alarmins, particularly interleukin (IL)-25, IL-33, and thymic stromal lymphopoietin (TSLP) (16–19). These cytokines trigger innate responses and prime the induction of an adaptive type-2 (Th2) immune response (16, 20, 21). They have also been established as essential to protection against infection with a number of model STHs (22–25). Epithelial cells themselves are potent reservoirs of these cytokines (26–28). Of recent interest is the role tuft cells play in sensing and responding to STH infection. Tuft cells are an epithelial cell subset that exist at low frequency during homeostasis but rapidly proliferate following STH infection (28–30). They sense the presence of STHs and intestinal microbes via taste-chemosensory receptors such as TRPM5 (29, 31) and secrete IL-25 and cysteinyl leukotrienes (CysLTs) to support the establishment of a Th2 mucosal response (28, 32).

Among the first lymphoid responders are the type-2 innate lymphoid cells (ILC2s). ILC2s have been shown to expand during STH infection and act as early sources of IL-4, IL-5 and IL-13 (33–35). Their depletion results in the delayed induction of Th2 immunity (33), although a non-redundant role for these cells in parasite expulsion has only been demonstrated for *N. brasiliensis* (36, 37). A broad role for alternative activation of macrophages (M2) has also been shown in most model STHs. M2s are required for the trapping and killing of the larval stages of *H. bakeri* and *N. brasiliensis* (38–40), this function is dependent on the production of arginase-1 (Arg-1) (41) and can be regulated by the expression level of resistin-like molecule (RELM)-α (40). Whilst expansion of other innate cells – including neutrophils, eosinophils, basophils, and mastocytes—at sites of infection is well-documented (42–44) a functional role for these cells in parasite expulsion has been harder to define and in some cases may be species-specific. For example, depletion of basophils is sufficient to trigger susceptibility to *T. muris* infection (45) but has no impact on resistance to *H. bakeri* (46). Similarly, mast cells and eosinophils have been linked to resistance to *H. bakeri* and *T. spiralis* (46, 47) but are redundant for expulsion of *T. muris* (48). Further, neutrophilia has been linked with expulsion of *N. brasiliensis* and *H. bakeri* (41, 42), via the release of neutrophil extracellular traps (NETs) (49) and support of M2 polarization (50). However, in cases where ablation of a given cell type does not result in a failure to attenuate infection, these cells may instead function to repair tissue damage once the infection has been resolved (51), or to moderate ongoing responses (40, 52). Alternatively, they may act to prime distal mucosal sites against future infection with other STH species, for example ILC2s primed by *T. spiralis* infection in the gut migrate to the lung and contribute to protection against a subsequent *N. brasiliensis* infection (53). Similarly, infection with *H. bakeri* results in protection against *N. brasiliensis* infection via IL-33-dependent induction of IL-5^+^CD4^+^ T cells capable of recruiting activated eosinophils to the lung (54).

Central to the expulsion of STHs is the CD4^+^ T cell. This can be inferred form studies of athymic nude mice which sustain long term high dose infections, compared to WT mice which readily expel parasites (55, 56). Depletion or ablation of CD4^+^ cells is enough to induce to susceptibility to infection in otherwise resistant mouse strains (15, 57). Further, adoptive transfer of CD4^+^ T cells to T and B cell deficient mice is sufficient to confer protection against infection (58). It is noteworthy that T cell deficient mouse strains such as athymic mice or recombinase 1 or 2 deficient mice still have a functional ILC2 compartment (36, 59, 60). A key function of CD4^+^ T cells is to provide Th2 cytokines—over and above those produced by ILC2s—in particular IL-4 and IL-13 which signal through IL-4 Receptor α (IL-4Rα) (61). IL-4Rα signaling drives a broad array of down-stream responses that are essential for the expulsion of STHs. These include; hyperproliferation of goblet cells (62); increased expression and secretion of mucins and anti-parasitic peptides, such as Muc5ac and RELM-β (63–66); increased turnover of epithelial cells (67, 68); enhanced gut contractility (69); immunoglobulin (Ig) class-switching to generate parasite-specific IgG1 (46, 70); and polarization of macrophages to an M2 phenotype (41, 71). The CD4^+^ T cell is also likely to be key to adaptive immune memory to STH infections. Under laboratory conditions, in immunocompetent mice, in response to a high-dose infection, these responses are robustly generated and lead to relatively rapid expulsion of the infection; although the kinetics differ based on genetic background of the host (39, 72–74).

In laboratory models of STH infection, as well as driving parasite expulsion, the immune response to a primary high-dose infection is sufficient to generate immunity to subsequent challenge infections (75–78). During secondary challenges the rate of expulsion is significantly accelerated. Depending on the species this may be a result of enhanced larval trapping mediated by parasite-specific IgG1 (46), priming of localized immune cells (50), or via expeditious induction of mucin secretion or mast cell activity. Regardless of mechanism, high-dose experimental infections produce robust sterilizing immunity to secondary infection.

## Natural STH Infection in Mammals

The artificially high doses given during experimental infections have proven a reliable system in which to investigate fundamental mechanisms of resistance to STH infection. However, this regime fails to reflect infection *in natura*. Not only is a single high-dose of parasites unrealistic in the wild, laboratory rodents are housed in pathogen free environments, with an abundance of resources, and a significant limit to stressors such as predators.

Experimental high-dose infections present a scenario in which primary STH infection is limited in duration, characterized by an immediate and potent Th2-polarized immune response, and generates sterilizing immunity to subsequent challenges. However, epidemiological evaluation of STH burden in human populations shows that in endemic areas, infected individuals suffer chronic parasitism throughout their lives (79–82). This holds true for non-human primates (83–85), livestock (86, 87), and wild rodent populations (88, 89). In humans, infection burden correlates strongly with age following one of two patterns: (i) parasite burden builds rapidly during early childhood but peaks shortly before adolescence, burden then declines and plateaus at a low level throughout adulthood e.g., *Trichuris trichiura*, and *Ascaris lumbricoides* (79, 90, 91); or (ii) STH burden builds consistently throughout childhood and early adolescence but plateaus at a moderate level prior to adulthood e.g., *Necator americanus* and *Ancylostoma duodenale* (82). Both patterns indicate that protective immunity to infection develops with age. However, they also suggest that this protection is incomplete and is preceded by a sustained period of susceptibility.

Our understanding of immune responses to STH infection *in natura* is limited. Much of what is known is founded on inferences drawn from blood samples taken from individuals living in endemic regions. As such, these data are caveated by an array of confounding factors. What is clear is that, in humans, up-regulation of the Th2 immune response is associated with STH infection. Importantly, Th2 associated markers, such as IgE and Th2 cytokines, show a clear negative correlation with worm burden (90, 91). Further, a strong IL-5 response in peripheral blood mononuclear cells (PBMC) isolated from infected individuals was shown to be predictive of resistance to reinfection following anthelminthic treatment (92, 93). A recent study utilizing mass cytometry to profile the Th2 and regulatory compartments before and after deworming in an Indonesian cohort confirmed a clear link between infection status and the expansion of ILC2s and Th2 cells, and reaffirmed the role of these cells in production of Th2 cytokines (94). Thus, a functional role for Th2 immunity in resistance to STH infection *in natura* is likely. What is interesting is that the development of this response is age-associated, with observed increases in anti-parasite IgE levels, and IL-13 and IL-4 production, in older individuals within the same cohort concurrent with a decrease in Th1-associated cytokines (90, 91, 95, 96). However, a complete polarization to Th2 immunity is rare, with most individuals maintaining a mixed Th1/Th2 response. The inability to generate a fully polarized protective response may be, in part, a consequence of STH-mediated immunosuppressive mechanisms. In one human cohort, deworming with the anthelmintic Albendazole resulted in an increase in STH-specific cytokine responses, and correlated with CD4^+^ T cells decreasing expression of the inhibitory molecule cytotoxic T lymphocyte-associated antigen 4 (CTLA-4) (97). A role for STH-controlled immune suppression in humans is supported by evidence that peptides derived from human-infecting hookworms (*Necator americanus* and *Ancylostoma duodenale*) are able to induce IL-10 and TGF-β signaling, and suppress IL-13 secretion in rodent models of colitis and allergy (98–100).

Age-associated, slowly developed resistance to infection is not unique to humans. Both feral and domestic sheep show progressive decreases in STH infection prevalence and fecal egg burden with age (101–103), as do domestic cattle (104). Wood mice (*Apodemus sylvaticus*) show an age-associated plateau in infection intensity of *H. polygyrus* akin to that seen in human hookworm infection (88). Further, non-human primates demonstrate slow acquisition of immunity to STHs following long periods of susceptibility, with the infection intensity kinetics of *T. trichiura* and other STHs paralleling those seen in humans (84, 85). Unfortunately, whilst the kinetics of these infections are broadly characterized, there is little in the way of immunological data accompanying these parasitological findings. However, it has been observed in wood mice that *H. polygyrus*-specific IgG1 titers increase with age and that treatment with the anthelminthics Ivermectin and Pyrantel was more effective in older mice relative to younger animals (105). This is consistent with a role for IgG1 in host-protection against *Heligmosomoides* (70).

Of significant importance to the outcome of infection is the overlapping geographical distribution of these parasites; not only with one another but also with other pathogens. STH-STH co-infections are highly common, and have been shown in a number of human cohorts to occur more frequently than single STH infections (106–108). In cases of STH-STH co-infection, infected individuals exhibit higher levels of infection for each individual species relative to individuals with a single-species infection (107, 108). From field data it remains unclear as to whether this is a correlative effect—i.e., an individual susceptible to infection with one species is simply more likely to be susceptible to infection with other STHs—or if STHs act synergistically by activating mechanisms that increase host susceptibility to infection. Experimental co-infection with *H. bakeri* and *T. muris*/*T. spiralis* has demonstrated that mice normally resistant to infection with *T. muris* or *T. spiralis* are rendered susceptible to infection when concurrently infected with *H. bakeri* (109, 110), although the mechanism through which this is mediated remains unresolved. Conversely, existing infection in the gut with *H. bakeri* or *T. spiralis* is protective against subsequent *N. brasiliensis* infection via priming of host-protective responses (53, 54). STH coinfection is also highly common with protist, bacterial and viral pathogens important to human health including malaria, tuberculosis and HIV. The primary focus of research into coinfections of this nature has been the effect STH coinfection has on the outcome of immune responses targeted the other pathogen. The overarching hypothesis being that as potent, and chronic, inducers of Th2/T-regulatory immunity STH infection will suppress the required Th1 immunity that targets single-cell/viral pathogens and thus increase susceptibility to infection and/or impact the efficacy of vaccines (111–113). However, it is also likely that infection with such pathogens will feedback onto the immune response against the STH. Therefore, it is important to bear in mind that *in natura* STH infections do not exist in isolation and have evolved in the context of a host immune system responding to a complex mix of co-infecting pathogens that elicit a diverse range of responses. Developing model systems with which to interrogate this reality presents exciting and challenging opportunities.

## Modeling Natural Infection

Given the difference in lifestyles experienced by laboratory rodents and their wild counterparts, it is perhaps unsurprising that there are considerable differences in their immune systems. Wild mice appear to exist in a state of higher immune activation with a more diverse repertoire of effector/effector-memory cells (114, 115) likely due to greater antigenic exposure (116). This may, in part, explain differences in observations between laboratory experiments, and natural exposure to STHs.

Few studies have attempted to experimentally mimic a “natural” setting for STH infection. Co-housing different mouse strains in large in-door enclosures and allowing for “natural” infection of *H. bakeri* (through contact with larvae in the enclosure as opposed to controlled oral administration) removed strain-specific resistance to infection resulting in longer-lived infections in BALB/c mice (117). Given the time period in which this experiment was conducted, the means through which this change in immune response occurred was not investigated. It could be speculated that co-housing mice on different backgrounds resulted in a change in the composition of the microbiome rendering previously resistant mice more susceptible to infection. It has previously been shown that strain specific resistance to infection by the enteric bacterial pathogen *Citrobacter rodentium* can be imposed on normally susceptible mice via fecal transfer from a resistant mouse strain; this effect was mediated by induction of host innate responses including IL-22-stimulated production of antimicrobial peptides (118, 119). However, whether a similar effect can be achieved with model STHs has yet to be shown.

More recently, C57BL/6 mice housed in controlled outdoor enclosures (a process known as “rewilding”) were shown to become susceptible to high-dose *T. muris* infection and exhibited impaired IL-13 production (120). Similar to observations in humans, higher worm burdens and biomass were correlated with reduced numbers of IL-13^+^CD4^+^ cells and increased frequency of IFNγ^+^CD4^+^ cells. The authors also found rewilding resulted in a marked increase in fecal microbial diversity. It will be of great interest to define the precise relationship between this increase in community diversity and the outcome of infection. In a subsequent analysis comparing uninfected mice housed in specific pathogen free (SPF) to those that were rewilded, it was shown that overall composition of blood and mesenteric lymph node immune cells was dramatically altered by the rewilding process including increases in central and effector memory T cells (121, 122). Interestingly, germ-free mice reconstituted with the caecal content of rewilded mice showed a significant increase in the proportion of granulocytes—in particular neutrophils—in the peripheral blood relative to mice reconstituted with caecal contents from SPF mice (121). Thus, rewilding has a profound and complex effect on immune cell composition, in part regulated by the microbiome, that may be responsible for impaired resistance to STH infection.

Together, these data suggest that inbred laboratory mice are not simply innately more resistant to STH infection than their wild counterparts, but that that environmental context is a major influence over the outcome of STH infection. Whilst studies that seek to recreate a more natural setting are valuable in bridging the gap between the laboratory and the field, they require an abundance of space and specialized facilities. They also re-introduce a myriad of confounding variables that reductionist laboratory model systems aim to nullify. Thus, the challenge is to develop an infection regime that is easily applicable to a traditional laboratory setting, recapitulates the dynamics observed in natural infections, and that limits the introduction of confounding factors.

## Trickle Infection

One factor that is easy to manipulate in a controlled fashion is the dose of parasites administered. In the *T. muris* system altering single infection dose within a single inbred strain of mouse is sufficient to change both resistance phenotype and the polarization of the immune response (123) with a high dose infection generating a Th2 response and acute infection and a single low dose, chronicity through the generation of a Th1 response (76). However, a single low-dose *T. muris* infection, in which a chronic infection characterized by a regulated Th1 response is established, also does not recapitulate the dynamic shift from susceptibility to a partial resistance, the observation generally seen in the field. The concept of “threshold” and “subthreshold” levels of infection associated with resistance or susceptibility is not new [see review by Behnke (124)]. Indeed, several observations from both natural and experimental STH infections in ruminants suggest that for some parasites—such as *Ostertagia ostertagia* (125), *Nematodirus battus* (126) and *Trichostrongylus sp* (127, 128)—“lower levels” of infection are consistent with longer survival of parasite burdens.

Historically, there has been interest in so-called “trickle” infections. In principal the trickle infection seeks to mimic natural exposure to a parasite by infecting animals with frequent low-doses of a given STH rather than a single high-dose.

Early on it was observed that, in rats, daily doses of five, third larval stage (L3) *N. brasiliensis* over either a 12 or 16 week period resulted in steady increase in worm numbers and egg output during the observation periods. Adult worm numbers at the end of the experiments were ≤ 30% of the total number of L3 administered. This data is, therefore, suggestive of partial immunity developing particularly in the latter stages of infection with stunted female worms containing reduced eggs numbers and the presence of few pre-adult/larval stages present. Indeed, if a large (50–1000 L3) single dose challenge infection was then administered to “trickled” rats, clear resistance was evident. When the trickle infection regime was increased to 50 L3 per day over 16 weeks, a rapid increase in parasite burden was observed that peaked at 2 weeks post infection, followed by a steady decline in infection intensity and an increase in the proportion of “stunted” adult female worms for the duration of experiment (129). The kinetics of this infection regime suggested that, in contrast to a high-dose infection in which a robust immune response would drive rapid parasite expulsion, the immune response developed slowly with repeated exposure and was only partially effective i.e., it limited subsequent infections but did not prevent their establishment. Although levels of infection differed, similar observations were made by Ovington (130). The immune response during trickle infections of *N. brasiliensis* has received little attention. With regards to peripheral antibody responses to parasite surface antigens, there were few differences between single dose and trickle infections (131). Ferens et al. (132, 133) using a shorter trickle of 10 infections of 25 L3 over a 4 week period, followed by a single large challenge, observed that trickle infections primed for a much more robust lung inflammation during the migratory phase of the infection through the lungs, than a single large dose priming infection. Bronchiolar lavage showed that trickle infection generated a marked elevation in eosinophils and alveolar macrophages. This may be indicative that trickle can effectively prime for robust immune mechanisms operating against pre-intestinal larval stages.

In concert with these observations, twice weekly trickle infection of 10–50 *H. bakeri* L3 in mice (134) or 30–50 *Ancylostoma ceylaniucum* larvae in hamsters (135) showed similar increases in worm burden followed by a steady decline. This slow expulsion of infection for both species was inhibited by treatment with cortisone (134, 135) suggesting immune control and induction of at least partial immunity by trickle infections. Again, in both systems, a high dose challenge after trickle was largely expelled although some worms still remained in the intestine.

A short-term trickle infection using *T. muris* demonstrated that infecting C57BL/6 mice on alternate days over the first 35 days of infection resulted an accumulation of parasites. Cytokine and serological analysis at the experimental end-point suggested that the trickle infected mice had an immunophenotype that was intermediate between mice that were infected with a Th2 polarizing high-dose infection and mice that had received a single low-dose infection known to drive a Th1 response (76). This intermediate phenotype parallels the immune-status of individuals in endemic regions that show a mixed Th1/Th2 response as opposed to the strong Th2 polarized responses seen in traditional experimental STH infections in rodents. However, trickle infection of Balb/K mice, a strain that is markedly more resistant than C57BL/6 to a single high dose infection, indicated that although trickle does lead to maintenance of worms within the intestine, lower levels of trickle were required to achieve this and this strain generated stronger Th2 response to the infection.

Similarly, CBA mice susceptible to *H. bakeri* infection had a blunted immune response during trickle infection and failed to initiate parasite expulsion compared to resistant SWR mice which were able to reduce their parasite burdens (136). Thus, as is evident with single-dose infections, genetic background can influence the progression of trickle infections.

Experimental trickle infections of *Trichuris suis* have also been undertaken in pigs. Pedersen and Saeed (137) used a trickle regime of 250 eggs twice weekly for 4 weeks and showed that substantial numbers of worms could be found in the gut at week 4 post infection although numbers were considerably reduced by week 14 (137). Trickled animals challenged at this point were significantly immune to a single high dose challenge infection. Nejsum et al. (138) used a more intense trickle regime administering at least 100 eggs per day over a 4, 8, or 14-week period, i.e., cumulative infections of ~ 4,000, 11,000, and 30,000 eggs, respectively. Significant numbers of worms (hundreds) were observed in the intestines at weeks 4 and 8, much lower than the numbest of eggs received. By week 14 few parasites were found in the intestine. Taken together, the data indicates immunity to *T. suis* can be built up after trickle infections over time and that the dynamics can be affected by the specific conditions of the trickle infection regime used.

We have recently reported a detailed characterization of a long-term trickle infection with *T. muris* (139). By performing weekly infections of 20 embryonated *T. muris* eggs in C57BL/6 mice we observed infection kinetics that closely mimicked those seen in human *T. trichiura* infection. Worm burden rose steadily with subsequent infections for 9 weeks, however, at 11 weeks we observed a decrease in worm burden and an absence of very early larval stages in the caeca of infected mice. This apparent acquisition of immunity correlated with an increase in Th2-associated immune responses including goblet cell hyperplasia, Muc5ac expression, and accelerated epithelial turnover (139). Importantly, depletion of CD4^+^ cells during the period of expulsion after trickle, removes protection. Given that these responses have been linked to resistance in previous studies using high-dose infection (66, 67, 140) it is reasonable to conclude that the modes by which resistance to *T. muris* are mediated are similar if not identical between single-dose and trickle infection. What is different between these modes of infection is the environment in which the initial response develops. In single high-dose infection immunologically naïve mice are a blank slate in which Th2 immunity can be rapidly generated. However, during trickle infection a Th2 response must develop in the context of an on-going Th1 response. Given that these types of immunity are mutually antagonistic, understanding the processes through which an on-going Th1 response transitions into a Th2-dominated state will be undoubtedly illuminating on fundamental mechanisms of immune regulation. Thus, a number of interesting questions present themselves.

### Is There a Set Threshold of Worm Burden That Can Be Tolerated Before Mechanisms of Expulsion Are Generated?

During trickle infection a transition occurs between susceptibility to infection and subsequent resistance to future challenges. In the case of *T. muris* this transition is dependent upon the number of doses, with fewer doses being insufficient to generate a protective immune response given the same exposure time (139). This would suggest that it is parasite burden, not the length of exposure that is essential in driving a shift in immune response. A dose-dependency in response to STHs can clearly be seen when comparing the outcome of single high-dose (acute, Th2-dominated) and low-dose (chronic, Th1 dominated) *T. muris* infection (123). However, what drives this polarization remains unclear. It is possible that there is a genetically set threshold of STH burden, influenced by local environment, that may be tolerated by the host beyond which point the cost exerted by the parasite becomes too great and must be reduced. One explanation could be that there exists a balance between the host response and STH-mediated immune-regulation designed to suppress Th2 immunity (141–143) and the effect of tissue damage that necessitates Th2-dependent wound repair (144). Were this the case, when the damaged caused by the STH becomes greater than its ability to immune-modulate the host, protective immunity is induced. These processes would be dynamic and change as number of infection events alter and the host responds. Recent work using single-dose *T. muris* infections has implicated B cells as important regulators of the balance between Th1/Th2 immunity. In BALB/c mice, which produce a potent Th2 response to a single high dose *T. muris*, infection antibody depletion of B cells had no effect. However, in C57BL/6 mice, which initially show a mixed Th1/Th2 response following infection, depletion of B cells resulted in an increase in Th1 cytokines, enhanced IFNγ-associated gene expression, and susceptibility to infection (145). This effect was antibody-independent and places B cells as potential regulators of IFNγ signaling during mixed Th responses. It is exciting to speculate that B cells, whilst previously thought to be largely dispensable for protection against *T. muris* (56, 57, 146), may play a role in tuning the Th response during trickle infection.

With each subsequent dose of parasite, as parasite burden increases, the relative concentration of available antigen is likely to rise proportionally. The effect of antigen load on T cell receptor (TCR) activation in STH infection remains poorly understood. Based on *in vitro* studies, using recombinant peptides not derived from STHs, it is canonically thought that a high level of TCR signaling, stimulated by higher antigen concentration or “quality,” favors Th1 differentiation whereas weaker signals allow for Th2 polarization (147). These observations were also mirrored *in vivo* (148) where it was speculated that paradoxically, large pathogens such as STH do not release amounts of antigen that readily gain access to antigen processing pathways, unlike rapidly dividing microbes. This may act in concert with immunomodulatory mechanisms employed by helminths. Antigen released from the eggs of the blood fluke *Schistosoma mansoni* actively reduces dendritic cell-T cell interactions lowering activation signal strength and directly biasing toward Th2 differentiation (149). This proactive induction of Th2 immunity by *S. mansoni* is thought to protect the host against severe pathology caused egg passage (150).

### Is There a Role for Tissue Damage in the Induction of Th2 Responses During Trickle Infection?

**Figure 1:**
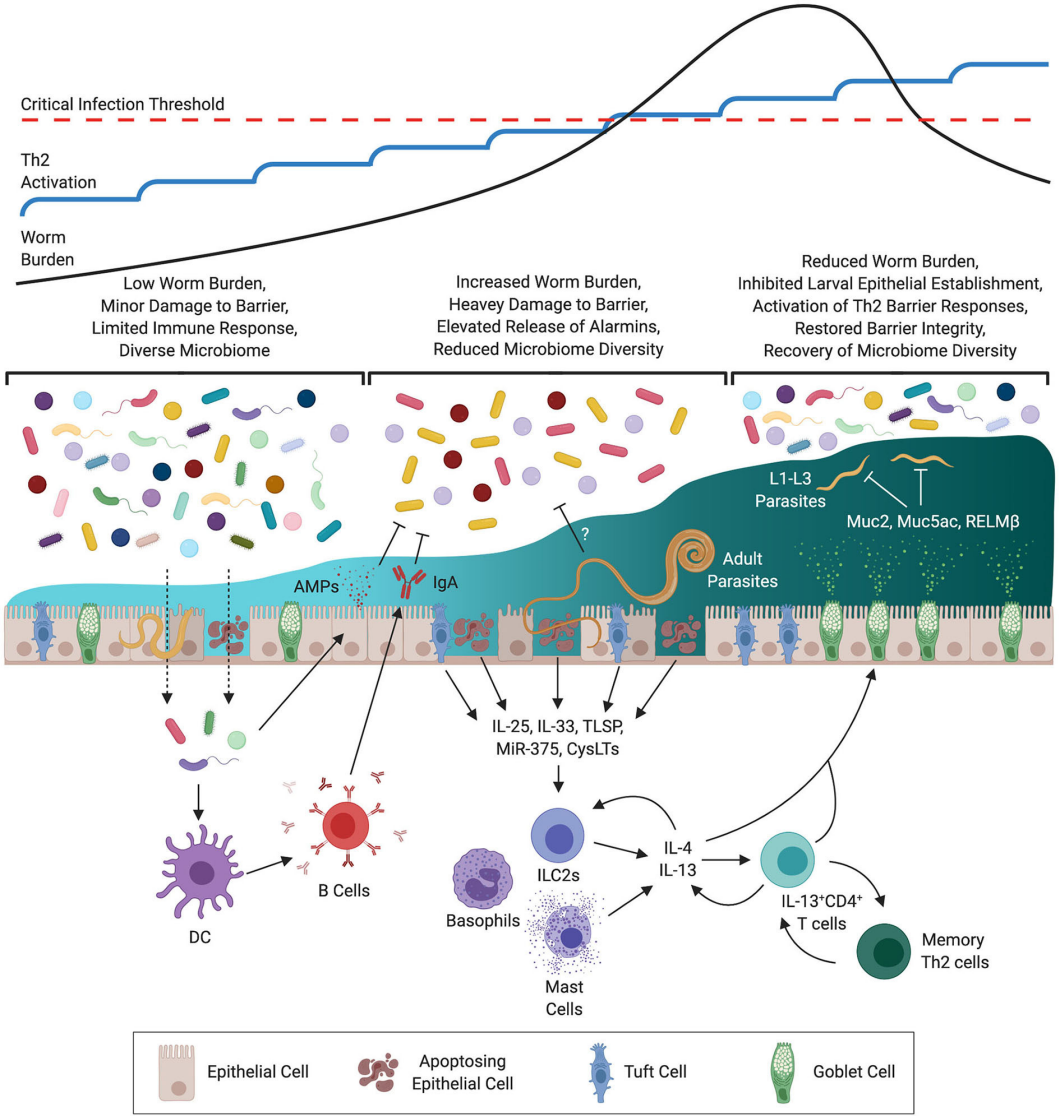
Development of Immunity During *Trichuris muris* Trickle Infection. At the outset of infection the low level of worm burden results in minor damage to the epithelial barrier. Whilst insufficient to drive a protective Th2 response this minor damage may be sufficient to allow for opportunistic invasion by commensal bacteria triggering the release of antimicrobial peptides (AMPs) and IgA. With repeated infections the level of barrier damage is exacerbated resulting in increased release of alarmins, micro RNAs (MiRs), and cysteinyl leukotrienes (CysLTs) from epithelial, mesenchymal, and innate cells. During this time a decrease in diversity of the microbiome is observed, this may be a result of immune-mediated regulation to prevent invasion by opportunistic pathogenic bacteria, or via STH-mediated remodeling. Activation of innate cells by type-2 signals results in the release of type-2 cytokines (IL-4 & IL-13) resulting in polarization of CD4^+^ T cells to a Th2 phenotype. Th2 cells then amplify the level of IL-4 & IL-13 signaling to activate host-protective responses at the epithelial barrier including goblet cell hyperproliferation, production of mucins such as Muc5ac, and heightened epithelial cell turnover. These responses operate primarily on early larval stages (L1–3) limiting the establishment of juvenile parasites within the epithelium. As a consequence barrier integrity is restored and intestinal microbial communities recover.

There is a well-established link between tissue damage and the type-2 immune response. As large macroparasites, STHs cause considerable damage upon invasion of the host and during the course of infection. Indeed, it has been argued that the responses to tissue damage and STH infection co-evolved so that mechanisms that facilitate parasite expulsion also mediate wound repair (144). Tissue damage results in epithelial, mesenchymal and innate-derived cytokines, capable of inducing Th2 responses, being released; these include TSLP, IL-25 and IL-33 (Figure 1) (25, 28, 151). Subsequent expansion of ILC2s, eosinophils, basophils, M2s and Th2 cells promotes/regulates both parasite expulsion and wound healing. As these cytokines are often produced as a result of cell damage, their concentrations present during infection are likely to reflect the magnitude of damage caused. Indeed, a role for IL-25 has been posited in late-stage expulsion of *H. bakeri* functioning as a key inducer of effector responses against adult-stage parasites (152). Therefore, during a trickle infection the concentration of these alarmins may increase with each subsequent challenge until a threshold concentration is reached that is sufficient to drive a protective response. This notion is consistent with the slow but progressive increase in Th2-associated responses observed during *T. muris* trickle (139). Further, damage-inducing microparticles have been previously shown to act as potent adjuvants capable of driving innate and antigen-specific Th2 immune responses *in vivo* comparable in efficacy to Alum (153, 154), as does mechanical abrasion (155). Epithelial derived micro(mi)RNAs are also known to influence resistance to STHs. Epithelial specific deletion of *Dicer*, a key gene encoding an RNAase involved in miRNA action, can change resistance to *T. muris* to susceptibility. MiR-375 was identified as an important miRNA in epithelial cells and deletion of MiR-375 in mice phenocopies the *Dicer* null response to *T. muris* (156). Little is known of the miRNA response to *T. muris* after trickle infections. A combination of the repeated release of alarmins, miRNAs and the Th2-specific adjuvant effect of tissue damage resulting from regular repeat infection may facilitate protective immunity.

### How Is an Effective Memory Response Generated During Trickle Infection?

Consistent with single-dose infection models there is an essential role for CD4^+^ T cells in immunity during trickle infection (139). These cells likely act as the dominant source of IL-13 that drives anti-parasite effector mechanisms. During infection, homing of T cells to the site of infection is essential for effective parasite expulsion (58, 157, 158). Following single high-dose STH infection, the memory T cells generated persist in the mucosa long after parasite expulsion and are sufficient to facilitate protection against subsequent challenge (159–161). However, the memory T cells generated following high-dose infection were primed in the context of a potent Th2 response, whereas memory cells generated during the early stages of a *T. muris* trickle infection will likely have been polarized to a Th1 phenotype. Moreover, the activity of the potentially pathogenic Th1 cells is regulated by IL-10 (162, 163) and thus regulation of ongoing responses also accompanies trickle infections. This scenario raises the question as to whether the CD4^+^ cells required for protective immunity in *T. muris* trickle infection arise from early memory T cells whose phenotype is plastic and informed by *de novo* production of Th2 cytokines by innate cells/environment, or if new CD4^+^ T cells are recruited later in infection? Repolarization of Th1 effector T cells into Th2 cells has previously been shown as a result of STH infection, OVA-specific Th1 cells transferred into naïve mice adopted a Th2 phenotype during *N. brasiliensis* infection (164), however, this was not in the context of an ongoing Th1 response. Instead, if naïve CD4^+^ T cells are recruited that differentiate into Th2 cells it will be interesting to determine if their TCR repertoire differs from Th1 cells generated early in infection. Identification of the specific antigens recognized by CD4^+^ T cells following the development of resistance under a trickle infection may provide a fertile avenue for the discovery of novel vaccine candidates.

### What Effect Does the Microbiome Have on the Outcome of Trickle Infection?

There exists an evident relationship between the immune system and the microbiome, especially in the gut where it is required for both the development and maintenance of the mucosal barrier (165), with loss of community diversity associated with inflammatory bowel disease (IBD) (166, 167). Whilst there are relatively few field studies that have investigated the relationship between STH infection and the microbiome in humans, it does appear that infection can affect microbial composition (168, 169). This is consistent with laboratory studies of chronic STH infection that have consistently shown that STHs alter the microbiome (170–172) and that these changes in composition can be reversed following expulsion of the infection (173). Chronic STH infection has been associated with expansion of bacterial genera with the capacity to promote the T regulatory response such as *Lactobacillus* (174–176) which may contribute to chronicity by suppressing the induction of a Th2 response. During the Th1-dominated susceptible phase of *T. muris* trickle infection there is a strong reduction of microbial diversity and an expansion of genera associated with chronic STH infection. Interestingly, the reduction in microbial diversity during the susceptible phase leads to a reduced efficiency of egg hatching (177) which is heavily dependent upon the intestinal microbiota (178). It can be speculated that this would have the net effect of keeping successive infection levels low, reducing the induction of protective immunity. Coinciding with the development of resistance during *T. muris* trickle infection the microbiome appears to partially recover with an increase in diversity and recovery of genera that had been lost earlier in infection (139). The nature of this relationship requires further assessment as several possibilities present themselves: (i) the development of a Th2 response actively promotes a homeostatic microbiota making the recovery in diversity a direct consequence of acquired immunity to STHs; (ii) recovery of the microbiome following loss of diversity occurs independently of host-driven mechanisms, but subsequently facilitates resistance by directly promoting a Th2 response; (iii) STHs produce antimicrobial peptides that restructure the microbiome to suit their own physiology, and when their numbers are reduced this effect is lost and the microbiome recovers as an indirect consequence of host-protective immunity (Figure 1).

## Concluding Remarks

Investigation of resistance and susceptibility to intestinal nematode parasites and their underlying immune mechanisms has not only informed on immunity to these particular infectious agents, but has identified novel and fundamental new information on how immunity works. This has no doubt arisen in part from the fact that infection by STHs present a particular set of challenges to the host immune system not seen in other pathogen infections.

The available evidence from the field and from experimental trickle infections of STH has led to a number of generally consistent core observations. Infection from exposure to a low number of infectious stages in any one infection event is more likely to lead to parasite patency than exposure to a “high” number of infectious stages in any one infection event (where the parasites are more often than not expelled, even if not completely). Thus, there appears to be a threshold for an infection event, below which the parasites do not get immunologically expelled and above which they do. This is not only influenced by host genetics, but also the local intestinal environment. It will also vary between different parasite species and the life cycle strategy that they have evolved. It is also clear that as long as the individual infection event remains below a certain level, increases in parasite load are tolerated up to a “critical point.” Again, number of infection events and interval between them will influence the ultimate success of the infection and the speed with which the “critical” point is reached. Ultimately, host protective immunity does begin to operate, although it is generally only partial and not sterilizing immunity. Adult parasites often remain for extended periods, although parasite fecundity eventually drops and new juvenile stages do not appear to be able to complete their development effectively i.e., are expelled. Thus, trickle infections are exemplars of concomitant immunity (179, 180). Protective immunological memory does occur with resistance to both high and low dose infection events, although some level of existing infection generally persists.

The single/challenge high dose infection approach to study experimental immunity to STH has been and continues to be spectacularly informative. Nevertheless, bearing in mind the way in which infections are acquired naturally, the trickle infection approach is set to further inform and refine our understanding of how protective immunity is generated, how it is regulated and, importantly, how it can be improved upon, especially for hosts that are naturally, chronically infected.

## Author Contributions

Both authors have made a substantial, direct and intellectual contribution to the work, and approved it for publication.

## Conflict of Interest

The authors declare that the research was conducted in the absence of any commercial or financial relationships that could be construed as a potential conflict of interest.
